# Flicker-induced retinal vasodilatation is not dependent on complement factor H polymorphism in healthy young subjects

**DOI:** 10.1111/aos.12433

**Published:** 2014-05-26

**Authors:** Reinhard Told, Stefan Palkovits, Agnes Boltz, Doreen Schmidl, Katarzyna J Napora, René M Werkmeister, Helmuth Haslacher, Sophie Frantal, Alina Popa-Cherecheanu, Leopold Schmetterer, Gerhard Garhöfer

**Affiliations:** 1 Department of Clinical Pharmacology, Medical University of ViennaVienna, Austria; 2 Center for Medical Physics and Biomedical Engineering, Medical University of ViennaVienna, Austria; 3 Department of Laboratory Medicine, Medical University of ViennaVienna, Austria; 4 Center for Medical Statistics, Informatics and Intelligence Systems, Medical University of ViennaVienna, Austria; 5 Department of Ophthalmology, Emergency University HospitalBucharest, Romania

**Keywords:** dynamic vessel analyzer, eye, flicker light stimulation, retinal circulation, Retinal vessel diameter, rs1061170, vasodilation, Y402H polymorphism, young healthy subjects

## Abstract

**Purpose:**

The complement factor H (CFH) tyrosine 402 histidine (Y402H, rs1061170) variant is known to be significantly associated with age-related macular degeneration (AMD). Whether this genetic variant may impact retinal blood flow regulation is largely unknown. This study investigated whether flicker-induced vasodilation, an indicator for the coupling between neural activity and blood flow, is altered in subjects carrying the rs1061170 risk allele.

**Methods:**

One hundred healthy subjects (aged between 18 and 45 years) were included in this study. Retinal blood flow regulation was tested by assessing retinal vessel calibres in response to stimulation with diffuse flicker light. Retinal vascular flicker responses were determined with a Dynamic Vessel Analyzer (DVA). In addition, genotyping for rs1061170 was performed.

**Results:**

Eighteen subjects were homozygous for the risk allele C, 50 were homozygous for the ancestral allele T, and 31 subjects were heterozygous (CT). One subject had to be excluded from data evaluation, as no genetic analysis could be performed due to technical difficulties. Baseline diameters of retinal arteries (p = 0.39) and veins (p = 0.64) were comparable between the three groups. Flicker-induced vasodilation in both retinal arteries (p = 0.38) and retinal veins (p = 0.62) was also comparable between the three studied groups.

**Conclusions:**

Our data indicate that homozygous healthy young carriers of the C risk allele at rs1061170 do not show abnormal flicker-induced vasodilation in the retina. This suggests that the high-risk genetic variant of CFH polymorphism does not impact neuro-vascular coupling in healthy subjects.

## Introduction

Alterations of the ocular circulation have been implicated in the pathogenesis of several ocular diseases, including age-related macular degeneration (AMD) (Feigl 2009), diabetic retinopathy (Pemp & Schmetterer 2008) and glaucoma (Grieshaber et al. 2007; Cherecheanu et al. 2013). Hemodynamic studies using different technologies, such as colour Doppler imaging (Ciulla et al. 1999), laser Doppler flowmetry (Grunwald et al. 1998) and ICG angiography (Ciulla et al. 2002), provide ample evidence of blood flow perturbations in patients with AMD in the choroidal and retrobulbar vasculature. In addition, it has been reported that lower choroidal perfusion is a risk factor for the development of choroidal neovascularization (CNV) in the fellow eye (Metelitsina et al. 2008; Boltz et al. 2010a,b).

Whereas the latter studies indicate that choroidal blood flow regulation is impaired in patients with AMD, less evidence is available concerning retinal blood flow regulation. A recent study, however, reports that retinal blood flow velocities are reduced in patients with AMD (Burgansky-Eliash et al. 2014). In addition, it has been shown that flickering light-induced vasodilation of retinal vessels is altered in patients with advanced AMD (Lanzl et al. 2011). Based on these findings, one might hypothesize that also retinal blood flow regulation may be compromised in patients with AMD.

We have recently shown that healthy carriers of the complement factor H polymorphism (CFH, rs1061170, Y402H) have altered choroidal blood flow autoregulation, even below 45 years (Told et al. 2013). This is of interest because AMD is closely linked to this CFH polymorphism (Boltz et al. 2010a,b). The Y402H polymorphism is located within the long arm of chromosome 1 (1q32) and encodes for the human CFH, a major complement regulatory protein, especially of the alternative pathway. Data from large epidemiological studies indicate that homozygous carriers of the CFH Y402H variant show a considerably increased risk for the development and progression of AMD and that this polymorphism may account for 50% of the attributable risk of AMD, suggesting that persons either homozygous or heterozygous for Y402H may account for one-half of AMD cases (Edwards et al. 2005). Similar results were found in various studies where the CFH polymorphism was present in over half of the included AMD patients (Schaumberg et al. 2006; Seddon et al. 2010; McKibbin et al. 2012; Hagstrom et al. 2013). Whether altered blood flow regulation, as observed in the choroid, is also present in the retinal circulation of high-risk carriers has not yet been investigated.

A potent way to non-invasively test retinal vascular function *in-vivo* is to assess flicker-light-induced changes of vascular calibres (Formaz et al. 1997; Riva et al. 2005). This provocation test is based on the observation that retinal vessels show a pronounced increase in vessel size when illuminated by flickering light at the frequency of 5–15 Hz. It is known that the vascular answer to flicker light stimulation is altered in patients with vascular disease (Kur et al. 2012) and that measurement of flicker-induced vasodilatation is an easily applicable tool to monitor functional microvascular alterations.

The current study was performed to investigate whether flicker-induced vasodilation is dependent on complement factor H polymorphism in healthy young subjects. This was carried out in an effort to clarify whether the regulation of retinal blood flow is dependent on complement factor H polymorphism.

## Materials and Methods

This study was performed in adherence to the Declaration of Helsinki and the Good Clinical Practice (GCP) guidelines. The study protocol was approved by the Ethics Committee of the Medical University of Vienna.

After written informed consent was obtained, 100 healthy subjects, aged between 18 and 45 years, were included in this study. The data presented in this report are part of a larger experiment, aiming at assessing choroidal autoregulation (Told et al. 2013). The same group of subjects was used in the present approach to study the impact of the rs1061170 polymorphism on retinal vessel response. Therefore, the sample size calculation was not based on flicker response, but on the main outcome of the project, which was choroidal blood flow (Told et al. 2013). As such, the sample size of the experiment was calculated based on the genotype frequency of the least occurring allele of the rs1061170 (homozygous risk allele carriers), which is approximately 14% in previous studies (Thakkinstian et al. 2006; Zee et al. 2006) and choroidal blood flow data (Garhofer et al. 2002).

### Experimental paradigm

Every subject was scheduled for a screening visit including medical history and physical examination. A blood sample was drawn for genetic analysis. Finally, an ophthalmic examination was performed, consisting of a visual acuity assessment with ETDRS charts, slit-lamp biomicroscopy, indirect fundoscopy and applanation tonometry. Inclusion criteria were normal ophthalmic and general findings unless the investigators considered an abnormality to be clinically irrelevant. Ametropia of more than three dioptres and anisometropia of more than one dioptre were exclusion criteria. In addition, subjects were excluded if they were smokers, took any type of medication or had a history of epilepsy.

On the study day, all measurements took place at the Department of Clinical Pharmacology, Medical University of Vienna. During a resting period of at least 20 min, the continuous stability of blood pressure and pulse rate was assured by repeated measurements in a sitting position. The dynamic vessel analyzer (DVA, Imedos, Jena, Germany) was used for retinal vessel analysis with flicker stimulation. Measurements started with 1 min of constant illumination of the fundus, followed by 1 min of flicker stimulation and ended with another minute of constant fundus illumination.

### Blood pressure and pulse rate

For systolic, diastolic and mean blood pressure levels, an automated oscillometric device was utilized (Wolzt et al. 1995). Blood pressure was assessed at the subjects’ upper arm. A finger pulse-oximetric device, which is attached to the monitor, was used to collect pulse rate data.

### Intraocular pressure

A slit-lamp-mounted Goldmann applanation tonometer was used to measure intraocular pressure (IOP) at prestudy screening and before and after flicker stimulation.

### Ocular perfusion pressure (OPP)

Ocular perfusion pressure was calculated as OPP = 2/3*MAP-IOP (Pournaras & Riva 2013).

### Genotyping of rs1061170

At the screening visit, a blood sample was drawn for genetic analysis. By the means of a Gentra® Puregene® Blood Kit (Qiagen GmbH, Hilden, Germany), DNA was extracted from EDTA-anticoagulated blood. The samples were stored at minus 80°C within the MedUni Vienna Biobank facility until measurements were taken. For genotyping, a real-time polymerase chain reaction (RT-PCR) on an ABI 7900HT Fast-Realtime thermocycler (Applied Biosystems, Rotkreuz, Switzerland) was performed applying sequence-specific, fluorescence-labelled TaqMan® probes with a minor groove binder and a non-fluorescent quencher.

Oligonucleotide sequences used were previously described by Goverdhan et al. (2008). 384-well plates with a total volume of 5 μl per reaction consisting of 2.5 μl TaqMan® Genotyping Mastermix (Applied Biosystems), 500 nm of each primer (VBC Genomics, Vienna, Austria), 200 nm of each probe (Applied Biosystems) and 10 ng DNA were used for RT-PCR. PCR parameters were 95°C for 10 min followed by 40 cycles of 15 seconds at 95°C and 1 min at 60°C. Finally, data were extracted using SDS 2.3 sequence detection software (Applied Biosystems).

### Dynamic vessel analyzer

The DVA is a commercially available system comprising a Zeiss FF 450 (Jena, Germany) fundus camera, a video camera as well as a video recording and analysing tool. Connected to a personal computer with specific software, the system allows recording of vessel diameters with 25 readings per second. The flicker frequency of the DVA system is 12.5 Hz, which has been shown to be in the range where maximum flicker excitation in human retinal vasculature can be achieved (Polak et al. 2002). The system provides excellent reproducibility and sensitivity (Polak et al. 2000; Garhofer et al. 2010).

Measurements of retinal vessel diameters were taken between 1 and 2 disc diameters from the margin of the optic disc in one inferior temporal artery and one inferior temporal vein.

### Data analysis

Subjects were grouped into three groups based on the results of the rs1061170 genotyping. The three resulting groups were homozygous risk allele carriers (C), homozygous ancestral gene carriers (T) and heterozygous (CT) subjects. Baseline (BL) values of vessel diameters were calculated as an average of the last 20 seconds before start of the flicker stimulation. Values during flicker (FL) stimulation were calculated as an average of the last 20 seconds of the stimulation period. Flicker-induced changes in retinal vessel diameters are expressed as percent of change over baseline values, that is (FL − BL) × 100/BL. The average percent of change was defined as the flicker response. A repeated-measures anova model was applied to test statistical significance. A p-value smaller than 0.05 was considered as statistically significant. CSS Statistica for Windows® (Statsoft Inc., Version 6.0, Tulsa, CA, USA) was used for all statistical calculations.

## Results

Of the 100 subjects screened, 18 were homozygous for the C allele, 50 were homozygous for the T allele, and 31 subjects were heterozygous allele carriers (CT, Table 1). One subject had to be excluded from the analysis, because genotyping was not possible due to technical difficulties.

**Table 1:** Baseline characteristics

	Homozygous C (*n* = 18)	Heterozygous CT (*n* = 31)	Homozygous T (*n* = 50)	p-Value
Age (years)	24.6 ± 4.9	24.8 ± 6.0	25.7 ± 6.4	0.70
Sex (M/F)	9/9	14/17	25/25	
MAP (mmHg)	80.3 ± 7.5	78.6 ± 8.2	80.1 ± 8.5	0.69
PR (beats per minute)	69.1 ± 11.7	72.4 ± 10.7	73.6 ± 9.8	0.29
IOP (mmHg)	14.1 ± 1.8	14.4 ± 2.2	14.8 ± 2.2	1.00
OPP (mmHg)	38.6 ± 5.3	38.8 ± 6.5	38.9 ± 5.4	0.98

Baseline demographical data and main characteristics of all subjects included are given in Table 1. As shown, no significant difference between the three groups was observed in terms of age, sex, mean arterial pressure (p = 0.87), pulse rate (p = 0.68), intraocular pressure (p = 1.0) and ocular perfusion pressure (p = 0.98).

**Figure 1:**
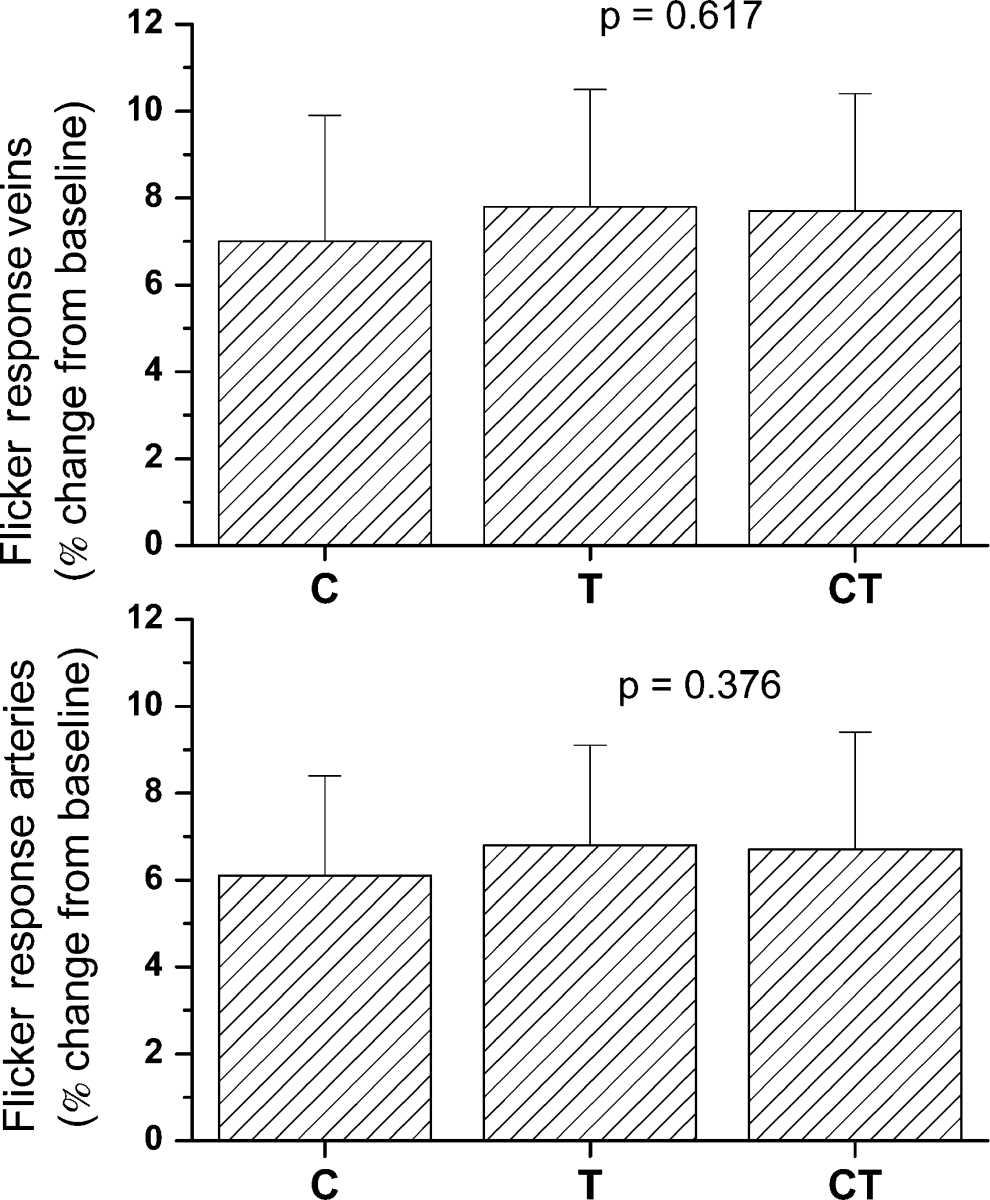
Retinal Vessel Flicker Response: Flicker response of retinal veins and arteries displayed as percent change from baseline diameter in homozygous risk allele carriers (C), ancestral gene carriers (T) and heterozygous subjects (CT).

No significant difference was found between the three groups in baseline arterial or venous vessel diameters. The baseline arterial diameters were 124.9 ± 12.8 μm, 122.0 ± 18.3 μm and 124.4 ± 14.4 μm in the risk allele carrier group (C), the ancestral gene carriers (T) and the heterozygous group, respectively (p = 0.39 anova between groups). The baseline venous diameter in the C, the T and the heterozygous group was 154.8 ± 16.7 μm, 153.1 ± 21.4 μm and 148.9 ± 17.4 μm (p = 0.64 anova between groups). Flicker stimulation induced a pronounced vasodilation in retinal veins and arteries in all three groups (Fig.1). Flicker stimulation induced an increase from baseline by 6.1 ± 2.3%, 6.8 ± 2.3% and 6.7 ± 2.7% (all p < 0.01 versus baseline) in the risk allele carrier group (C), the ancestral gene carriers (T) and the heterozygous group, respectively. Retinal venous diameters increased after flicker stimulation by 7.0 ± 2.9%, 7.8 ± 2.7% and 7.7 ± 2.7% in the C, T and heterozygous group, respectively (all p < 0.01 versus baseline). As shown in Fig.1, no significant difference was observed between the three groups in terms of flicker response in retinal arteries or retinal veins (arteries p = 0.38 anova between groups, veins p = 0.62 anova between groups).

## Discussion

Retinal vascular dysregulation has been observed in a variety of ocular diseases such as diabetic retinopathy or glaucoma (Pemp & Schmetterer 2008; Pournaras et al. 2008; Venkataraman et al. 2010; Kur et al. 2012; Pournaras & Riva 2013). As such, it has been shown that flicker-induced vasodilation is reduced in early- and late-stage diabetic retinopathy (Garhofer et al. 2004a,b,c; Mandecka et al. 2007; Nguyen et al. 2009a,b; Pemp et al. 2009; Lasta et al. 2013), in patients with early-stage glaucoma (Garhofer et al. 2004a,b,c; Gugleta et al. 2012) and in patients with systemic hypertension (Nagel et al. 2004).

However, whether vascular dysregulation is also present in the retinal circulation of patients with AMD is still a matter of controversy. Whereas Sato and colleagues (Sato et al. 2006) did not find a difference in mean blood velocity, arterial diameter or mean blood flow rate in patients with AMD, a more recent study demonstrates reduced blood flow velocities in patients with AMD compared with healthy subjects (Burgansky-Eliash et al. 2014). Human studies investigating flicker-induced vasodilation in patients with AMD have also reported an altered flicker response in subjects with advanced AMD (Lanzl et al. 2011). Whether these results can be attributed to vascular dysregulation leading to a decreased sensitivity of the retina to the flicker stimulus or to neuronal dysfunction remains unclear. A study using the focal electroretinogram (fERG) revealed a significant impact of the rs1061170 polymorphism on retinal function in patients with early AMD (Capoluongo et al. 2012). In particular, this study shows that mean fERG amplitude and sensitivity decreased in patients carrying the risk allele compared with ancestral gene carriers although visual acuity and funduscopic features were comparable (Capoluongo et al. 2012).

To the best of our knowledge, this study is the first to investigate whether genetic polymorphisms determine flicker-induced vasodilation in young healthy subjects. Our data indicate that a common polymorphism in the complement factor H gene that is associated with AMD does not affect the retinal vascular response to stimulation with diffuse luminance flicker. No difference was found; neither the venous nor the arterial flicker response was altered comparing homozygous risk allele carriers (C), ancestral gene carriers (T) and the heterozygous group.

As mentioned above, we have recently shown that the response of the choroidal circulation to an increase in ocular perfusion pressure (OPP) as induced by isometric exercise takes place at lower OPP levels in healthy subjects homozygous for the rs1061170 C variant compared with ancestral allele carriers and heterozygous subjects (Told et al. 2013). This indicates that in subjects carrying the risk allele, the choroidal blood flow response to choroidal perfusion pressure is altered, even in young subjects (Told et al. 2013). This is also in good agreement with a Finnish study indicating that this polymorphism is associated with altered carotid artery elasticity in healthy young subjects (Jylhava et al. 2009). The reason for this impaired regulation process is not entirely clear. It has been hypothesized that these findings relate to subclinical inflammatory processes occurring already very early in life of these patients (Machalinska et al. 2012; Told et al. 2013). Along this line of thought, it has been shown that variants of this polymorphism increase the risk of myocardial infarction (Kardys et al. 2006) and are involved in the pathogenesis of atherosclerosis (Oksjoki et al. 2003) and inflammatory processes (Haines et al. 2005). In addition, CFH-deficient mice show C3 and C3b complement protein deposition at endothelial cells in the choroidal and retinal vasculature leading to narrowing and dying out of vessels with subsequent ischaemia (Lundh von Leithner et al. 2009).

It is interesting to note that in contrast to the choroidal circulation, where blood flow regulation is dependent on complement factor H polymorphisms (Told et al. 2013), no differences in flicker-induced vasodilation of retinal vessels in carriers of different risk alleles were observed. It has, however, to be considered that the retina and the choroid are two vascular beds with different physiological properties. Whereas the retina represents a vascular bed highly regulated in response to local metabolic demands, the choroidal circulation is regulated to a large degree by neural mechanisms. Additionally, flicker-induced retinal vasodilation and the autoregulatory response of the choroid are based on different physiological mechanisms reflecting the distinct mechanisms that control blood flow in the two vascular beds (Pournaras et al. 2008; Schmidl et al. 2011). Stimulation with flickering light leads to vasodilation and causes local hyperaemia (Garhofer et al. 2004a,b,c), induced by an increase in local metabolic demands due to augmented neural activity (Riva 2006; Kur et al. 2012). In contrast to that the stimuli for the regulatory answer of the choroid in face of an increase in perfusion pressure are caused by a vasoconstrictor response to maintain choroidal perfusion (Cherecheanu et al. 2013). Considering these differences, it seems reasonable that complement factor H polymorphism shows a different effect on the retinal circulation compared with the choroidal circulation.

Previous studies, however, have reported that also retinal vessel diameters are at least partially genetically determined (Taarnhoj et al. 2006; Xing et al. 2006). Data from the Beaver Dam Eye study revealed that a number of different genes and loci are linked to retinal vessel development and retinal vessel diameters (Xing et al. 2006). Whether the rs1061170 polymorphism is related with differences in retinal vessel calibres, is, however, still a matter of controversy (Xing et al. 2006) and goes beyond the scope of the present study. No significant differences were found between retinal arterial or venous baseline diameters in the three study groups. One needs, however, to consider that not all retinal vessels were analysed in the present study.

Although the current study found a tendency towards a decrease in flicker-induced vasodilation, no significant difference was found between the risk allele carriers and the other groups. Given that only 18 subjects were homozygous carriers of the risk allele, the power of the study might be too weak to detect small differences in flicker response. Here, one also needs to mention that our sample size calculation was based on the choroidal blood flow experiments. Based on the data gained in the current study and previous reproducibility data using the same technique (Garhöfer G, Told R, Boltz A, Schmidl D, Palkovits S, Schmetterer L, unpublished data), post hoc power estimation reveals that our study was capable to detect differences in the flicker response between groups of 35% or more. This is based on a power of 80% and an alpha error of 5%. Thus, we cannot exclude that we may have missed differences in retinal flicker response smaller than 35%.

As a limitation of the current study, it needs to be mentioned that the DVA is not capable of measuring retinal vessels of a size of 90 μm or smaller (Garhofer et al. 2009). It is, however, known that precapillary arterioles exert a major contribution in resistance to flow and play therefore a major role in the regulation of blood flow. Considering the pronounced increase of more than 50% in volumetric blood flow induced by flicker stimulation (Garhofer et al. 2004a) together with the only moderate vasodilation observed in major retinal vessels, it seems reasonable to suggest that most of the dilatory response occurs in the microcirculation. Unfortunately, vessels in the size of small arterioles and precapillary arterioles are not accessible with standard instruments such as the DVA. The DVA determines vessel diameters based on the differences in the brightness profile of the erythrocyte column within the vessel compared with the surrounding. Thus, the resolution of the technique is limited by optical errors of the eye but also by technical limitations such as the resolution of the fundus camera and the connected video system.

In conclusion, the data of the current study do not support the hypothesis that retinal vascular regulation in response to neuronal stimulation is influenced by the CFH polymorphism rs1061170 as it is observed for the choroid during increase in OPP. Whether this is related to the different stimuli used, to the differences in the vascular beds or to other reasons remains unclear.
